# 3-Isopropyl-2-(4-methoxy­phen­oxy)-1-benzo­furo[3,2-*d*]pyrimidin-4(3*H*)-one

**DOI:** 10.1107/S1600536809042925

**Published:** 2009-10-23

**Authors:** Xiao-Bao Chen, Jing Xu, Ai-Hua Zheng, Jia-Hua Tian, Hong Luo

**Affiliations:** a Institute of Medicinal Chemistry, Yunyang Medical College, Shiyan, Hubei 442000, People’s Republic of China

## Abstract

In the title compound, C_20_H_18_N_2_O_4_, all non-H atoms of the three fused rings of the benzofuro[3,2-*d*]pyrimidine system are almost coplanar (r.m.s. deviation 0.021 Å). The dihedral angle between the fused ring system and the benzene ring is 1.47 (12)°. Intra­molecular and inter­molecular C—H⋯O hydrogen bonds together with weak C—H⋯π inter­actions stabilize the structure.

## Related literature

For the biological activity of benzofuropyrimidine derivatives, see: Bodke & Sangapure (2003 ▶). For the synthesis of the title compound, see: Ding *et al.* (2004 ▶). For the structures of other fused pyrimidinone derivatives, see: Hu *et al.* (2005 ▶, 2006 ▶, 2007 ▶).
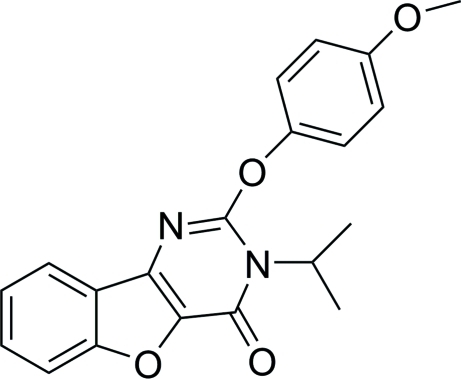


## Experimental

### 

#### Crystal data

C_20_H_18_N_2_O_4_
*M* *_r_* = 350.36
Monoclinic, 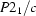

*a* = 10.0358 (7) Å
*b* = 14.2879 (10) Å
*c* = 13.2071 (9) Å
β = 112.089 (1)°
*V* = 1754.8 (2) Å^3^
*Z* = 4
Mo *K*α radiation
μ = 0.09 mm^−1^
*T* = 298 K
0.26 × 0.13 × 0.10 mm

#### Data collection

Bruker SMART CCD area-detector diffractometer
Absorption correction: none
11156 measured reflections
3809 independent reflections
3354 reflections with *I* > 2σ(*I*)
*R*_int_ = 0.040

#### Refinement

*R*[*F* ^2^ > 2σ(*F* ^2^)] = 0.070
*wR*(*F* ^2^) = 0.155
*S* = 1.22
3809 reflections
238 parameters
H-atom parameters constrained
Δρ_max_ = 0.19 e Å^−3^
Δρ_min_ = −0.23 e Å^−3^

### 

Data collection: *SMART* (Bruker, 2001 ▶); cell refinement: *SAINT* (Bruker, 2001 ▶); data reduction: *SAINT*; program(s) used to solve structure: *SHELXS97* (Sheldrick, 2008 ▶); program(s) used to refine structure: *SHELXL97* (Sheldrick, 2008 ▶); molecular graphics: *PLATON* (Spek, 2009 ▶); software used to prepare material for publication: *SHELXTL* (Sheldrick, 2008 ▶).

## Supplementary Material

Crystal structure: contains datablocks global, I. DOI: 10.1107/S1600536809042925/bt5104sup1.cif

Structure factors: contains datablocks I. DOI: 10.1107/S1600536809042925/bt5104Isup2.hkl

Additional supplementary materials:  crystallographic information; 3D view; checkCIF report

## Figures and Tables

**Table 1:** Hydrogen-bond geometry (Å, °)

*D*—H⋯*A*	*D*—H	H⋯*A*	*D*⋯*A*	*D*—H⋯*A*
C4—H4⋯O4^i^	0.93	2.49	3.311 (3)	147
C11—H11⋯O2	0.98	2.25	2.761 (3)	111
C12—H12*C*⋯O3	0.96	2.32	2.845 (4)	114
C13—H13*A*⋯O3	0.96	2.41	2.957 (3)	116
C16—H16⋯*Cg*2^ii^	0.93	2.76	3.551 (2)	143
C19—H19⋯*Cg*3^iii^	0.93	2.90	3.742 (3)	152

Symmetry codes: (i) 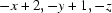; (ii) 
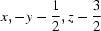
; (iii) 
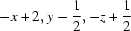
. *Cg*2 and *Cg*3 are the centroids of the N1/C8/C7/C10/N2/C9 and C1–C6 rings, respectively.

**Fig. 1.**
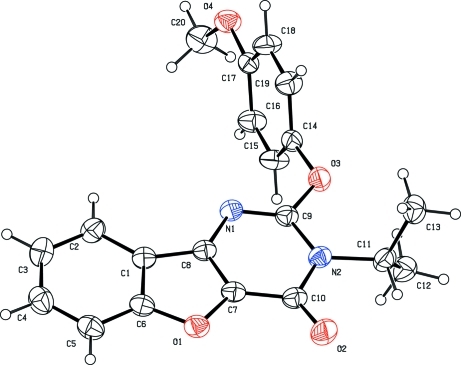
View of the molecular structure of (I), showing the atom labelling schemeand with displacement ellipsoids drawn at the 50% probability level.

**Fig. 2.**
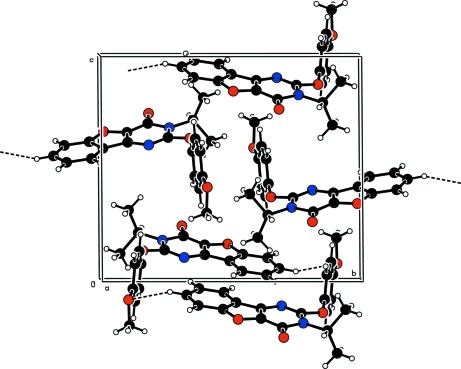
A partial view of the crystal packing of (I), showing the formation of C—H···O hydrogen-bonded. showing as dashed lines.

## supplementary crystallographic information

### Comment

Benzofuropyrimidine derivatives are of interest as possible antiviral agents, and because of their other biological properties, including antibacterial, antifungal, antiallergic and antiinflammatory activities (Bodke & Sangapure, 2003). We have recently focused on the synthesis of the fused heterocyclic systems containing pyrimidinone *via* aza-Wittig reactions at room temperature (Ding *et al.*, 2004). We present here the structure of such a benzofuropyrimidinone derivative. Fig. 1 shows the molecular structure of the title compound with the atomic numbering scheme. Intramolecular C—H···O and intermolecular C—H···O hydrogen bonds together with weak C—H···π interactions (Table 1) stabilize the structure. (Fig.2).

### Experimental

To a solution of *N*-(2-ethoxycarbonylbenzofuran-3-yl)iminotriphenylphosphorane (3 mmol) in dry dichloromethane (15 ml) was added isopropyl isocyanate (3 mmol) under nitrogen at room temperature. After the reaction mixture had been allowed to stand for 20 h at 273–278 K, the solvent was removed under reduced pressure and diethyl ether-petroleum ether (1:2 *v*/*v*, 20 ml) was added to precipitate the triphenylphosphine oxide. After filtration, the solvent was removed to give the ethyl 3-((isopropylimino)methyleneamino)-2,3-dihydrobenzofuran-2-carboxylate, which was used directly without further purification. To a solution of ethyl 3-((isopropylimino)methyleneamino)-2,3- dihydrobenzofuran-2-carboxylate in acetonitrile (15 ml) were added 4-methylphenol (3 mmol) and anhydrous K_2_CO_3_ (1 mmol). The mixture was stirred for 6 h at 313–323 K. The solution was concentrated under reduced pressure and the residue was recrystallized from dichloromethane and ethanol (1:2 *v*/*v*) to give the title compound. Suitable crystals were obtained by vapour diffusion of ethanol into dichloromethane at room temperature.

### Refinement

H atoms  were placed at calculated positions, with C—H distances of 0.97 and 0.93Å for H atoms bonded to *sp*^3^ and *sp*^2^ C atoms, respectively. They were refined using a riding model, with *U*_iso_(H) = 1.2*U*_eq_(C)  or 1.5*U*_eq_(methyl C).

### Crystal data

**Table:**

C_20_H_18_N_2_O_4_	*F*(000) = 736
*M**_r_* = 350.36	*D*_x_ = 1.326 Mg m^−^^3^
Monoclinic, *P*2_1_/*c*	Mo *K*α radiation, λ = 0.71073 Å
*a* = 10.0358 (7) Å	Cell parameters from 4035 reflections
*b* = 14.2879 (10) Å	θ = 2.2–27.2°
*c* = 13.2071 (9) Å	µ = 0.09 mm^−^^1^
β = 112.089 (1)°	*T* = 298 K
*V* = 1754.8 (2) Å^3^	Block, colorless
*Z* = 4	0.26 × 0.13 × 0.10 mm

### Data collection

**Table:**

Bruker SMART CCD area-detector diffractometer	3354 reflections with *I* > 2σ(*I*)
Radiation source: fine-focus sealed tube	*R*_int_ = 0.040
graphite	θ_max_ = 27.0°, θ_min_ = 2.2°
phi and ω scans	*h* = −12→12
11156 measured reflections	*k* = −14→18
3809 independent reflections	*l* = −16→16

### Refinement

**Table:**

Refinement on *F*^2^	Primary atom site location: structure-invariant direct methods
Least-squares matrix: full	Secondary atom site location: difference Fourier map
*R*[*F*^2^ > 2σ(*F*^2^)] = 0.070	Hydrogen site location: inferred from neighbouring sites
*wR*(*F*^2^) = 0.155	H-atom parameters constrained
*S* = 1.22	*w* = 1/[σ^2^(*F*_o_^2^) + (0.0482*P*)^2^ + 0.6117*P*] where *P* = (*F*_o_^2^ + 2*F*_c_^2^)/3
3809 reflections	(Δ/σ)_max_ < 0.001
238 parameters	Δρ_max_ = 0.19 e Å^−^^3^
0 restraints	Δρ_min_ = −0.23 e Å^−^^3^

### Special details

**Table:**

Geometry. All e.s.d.'s (except the e.s.d. in the dihedral angle between two l.s. planes) are estimated using the full covariance matrix. The cell e.s.d.'s are taken into account individually in the estimation of e.s.d.'s in distances, angles and torsion angles; correlations between e.s.d.'s in cell parameters are only used when they are defined by crystal symmetry. An approximate (isotropic) treatment of cell e.s.d.'s is used for estimating e.s.d.'s involving l.s. planes.
Refinement. Refinement of *F*^2^ against ALL reflections. The weighted *R*-factor *wR* and goodness of fit *S* are based on *F*^2^, conventional *R*-factors *R* are based on *F*, with *F* set to zero for negative *F*^2^. The threshold expression of *F*^2^ > σ(*F*^2^) is used only for calculating *R*-factors(gt) *etc*. and is not relevant to the choice of reflections for refinement. *R*-factors based on *F*^2^ are statistically about twice as large as those based on *F*, and *R*- factors based on ALL data will be even larger.

### Fractional atomic coordinates and isotropic or equivalent isotropic displacement parameters (Å^2^)

**Table:**

	*x*	*y*	*z*	*U*_iso_*/*U*_eq_	
C1	0.7439 (2)	0.49152 (15)	0.08888 (16)	0.0449 (5)	
C2	0.8316 (3)	0.53714 (18)	0.04468 (19)	0.0556 (6)	
H2	0.8998	0.5043	0.0268	0.067*	
C3	0.8149 (3)	0.63207 (19)	0.0280 (2)	0.0665 (7)	
H3	0.8725	0.6640	−0.0016	0.080*	
C4	0.7126 (3)	0.68112 (18)	0.0549 (2)	0.0649 (7)	
H4	0.7023	0.7451	0.0413	0.078*	
C5	0.6270 (3)	0.63811 (17)	0.1005 (2)	0.0588 (6)	
H5	0.5600	0.6713	0.1194	0.071*	
C6	0.6450 (2)	0.54273 (16)	0.11700 (17)	0.0484 (5)	
C7	0.6210 (2)	0.39759 (16)	0.15826 (17)	0.0453 (5)	
C8	0.7248 (2)	0.39635 (15)	0.11576 (16)	0.0428 (5)	
C9	0.7502 (2)	0.24186 (15)	0.13723 (17)	0.0440 (5)	
C10	0.5734 (2)	0.31676 (17)	0.19732 (17)	0.0480 (5)	
C11	0.6003 (2)	0.14330 (17)	0.21223 (19)	0.0523 (6)	
H11	0.5180	0.1570	0.2325	0.063*	
C12	0.5457 (3)	0.0739 (2)	0.1184 (2)	0.0671 (7)	
H12A	0.4782	0.1044	0.0552	0.101*	
H12B	0.4994	0.0226	0.1389	0.101*	
H12C	0.6251	0.0508	0.1019	0.101*	
C13	0.7157 (3)	0.10533 (19)	0.3141 (2)	0.0654 (7)	
H13A	0.8000	0.0910	0.2992	0.098*	
H13B	0.6815	0.0495	0.3369	0.098*	
H13C	0.7390	0.1513	0.3712	0.098*	
C14	0.9024 (2)	0.14978 (15)	0.07749 (18)	0.0448 (5)	
C15	0.8475 (2)	0.14131 (19)	−0.0328 (2)	0.0605 (6)	
H15	0.7490	0.1470	−0.0714	0.073*	
C16	0.9378 (2)	0.12422 (18)	−0.08780 (19)	0.0567 (6)	
H16	0.9002	0.1187	−0.1635	0.068*	
C17	1.0832 (2)	0.11536 (14)	−0.03082 (18)	0.0438 (5)	
C18	1.1364 (2)	0.12380 (19)	0.08137 (19)	0.0601 (6)	
H18	1.2347	0.1177	0.1207	0.072*	
C19	1.0466 (2)	0.14111 (19)	0.13570 (19)	0.0574 (6)	
H19	1.0834	0.1469	0.2114	0.069*	
C20	1.1334 (3)	0.1004 (2)	−0.1929 (2)	0.0735 (8)	
H20A	1.0674	0.0498	−0.2234	0.110*	
H20B	1.2143	0.0941	−0.2144	0.110*	
H20C	1.0859	0.1589	−0.2192	0.110*	
N1	0.79382 (18)	0.31642 (13)	0.10474 (14)	0.0452 (4)	
N2	0.64378 (18)	0.23540 (13)	0.17913 (14)	0.0456 (4)	
O1	0.56950 (16)	0.48638 (11)	0.16127 (13)	0.0528 (4)	
O2	0.48616 (19)	0.31249 (13)	0.24071 (16)	0.0677 (5)	
O3	0.80979 (17)	0.15842 (11)	0.13495 (14)	0.0561 (4)	
O4	1.18117 (17)	0.09783 (12)	−0.07769 (13)	0.0584 (4)	

### Atomic displacement parameters (Å^2^)

**Table:**

	*U*^11^	*U*^22^	*U*^33^	*U*^12^	*U*^13^	*U*^23^
C1	0.0430 (11)	0.0496 (12)	0.0382 (11)	−0.0016 (9)	0.0107 (9)	−0.0053 (9)
C2	0.0547 (13)	0.0561 (15)	0.0600 (14)	−0.0039 (11)	0.0262 (11)	−0.0047 (11)
C3	0.0730 (17)	0.0602 (16)	0.0691 (17)	−0.0135 (13)	0.0298 (14)	−0.0014 (13)
C4	0.0740 (17)	0.0493 (14)	0.0637 (16)	−0.0016 (12)	0.0171 (13)	−0.0009 (12)
C5	0.0604 (14)	0.0517 (14)	0.0591 (15)	0.0087 (11)	0.0165 (12)	−0.0055 (11)
C6	0.0458 (11)	0.0535 (13)	0.0413 (11)	0.0041 (10)	0.0112 (9)	−0.0028 (9)
C7	0.0404 (11)	0.0526 (13)	0.0428 (11)	0.0062 (9)	0.0154 (9)	−0.0020 (9)
C8	0.0387 (10)	0.0508 (12)	0.0369 (10)	0.0011 (9)	0.0121 (8)	−0.0028 (9)
C9	0.0417 (11)	0.0494 (12)	0.0416 (11)	0.0044 (9)	0.0165 (9)	0.0009 (9)
C10	0.0412 (11)	0.0598 (14)	0.0440 (12)	0.0039 (10)	0.0172 (9)	−0.0007 (10)
C11	0.0488 (12)	0.0560 (14)	0.0592 (14)	−0.0052 (10)	0.0283 (11)	0.0011 (11)
C12	0.0556 (14)	0.0657 (16)	0.0727 (17)	−0.0083 (12)	0.0159 (13)	−0.0067 (13)
C13	0.0754 (17)	0.0685 (17)	0.0530 (14)	−0.0054 (13)	0.0250 (13)	0.0105 (12)
C14	0.0465 (11)	0.0387 (11)	0.0533 (13)	0.0051 (9)	0.0236 (10)	0.0039 (9)
C15	0.0395 (11)	0.0805 (18)	0.0561 (15)	0.0139 (11)	0.0118 (10)	0.0019 (12)
C16	0.0485 (12)	0.0753 (17)	0.0423 (12)	0.0080 (11)	0.0126 (10)	−0.0022 (11)
C17	0.0438 (11)	0.0368 (11)	0.0532 (12)	0.0047 (8)	0.0210 (9)	0.0034 (9)
C18	0.0355 (11)	0.0880 (19)	0.0519 (14)	0.0053 (11)	0.0109 (10)	0.0042 (12)
C19	0.0516 (13)	0.0754 (17)	0.0435 (12)	0.0023 (12)	0.0158 (10)	0.0002 (11)
C20	0.0799 (18)	0.088 (2)	0.0628 (17)	0.0156 (15)	0.0391 (15)	−0.0024 (14)
N1	0.0425 (9)	0.0485 (10)	0.0489 (10)	0.0028 (8)	0.0220 (8)	−0.0005 (8)
N2	0.0415 (9)	0.0539 (11)	0.0432 (10)	−0.0009 (8)	0.0179 (8)	0.0001 (8)
O1	0.0511 (9)	0.0542 (9)	0.0589 (10)	0.0086 (7)	0.0272 (8)	−0.0022 (7)
O2	0.0671 (11)	0.0722 (12)	0.0841 (12)	0.0062 (9)	0.0517 (10)	0.0032 (9)
O3	0.0616 (10)	0.0494 (9)	0.0714 (11)	0.0106 (7)	0.0411 (8)	0.0114 (8)
O4	0.0529 (9)	0.0666 (11)	0.0617 (10)	0.0120 (8)	0.0285 (8)	0.0043 (8)

### Geometric parameters (Å, °)

**Table:**

C1—C2	1.388 (3)	C11—H11	0.9800
C1—C6	1.392 (3)	C12—H12A	0.9600
C1—C8	1.436 (3)	C12—H12B	0.9600
C2—C3	1.374 (4)	C12—H12C	0.9600
C2—H2	0.9300	C13—H13A	0.9600
C3—C4	1.395 (4)	C13—H13B	0.9600
C3—H3	0.9300	C13—H13C	0.9600
C4—C5	1.367 (4)	C14—C15	1.355 (3)
C4—H4	0.9300	C14—C19	1.365 (3)
C5—C6	1.381 (3)	C14—O3	1.409 (2)
C5—H5	0.9300	C15—C16	1.380 (3)
C6—O1	1.377 (3)	C15—H15	0.9300
C7—C8	1.357 (3)	C16—C17	1.373 (3)
C7—O1	1.376 (3)	C16—H16	0.9300
C7—C10	1.418 (3)	C17—O4	1.368 (2)
C8—N1	1.372 (3)	C17—C18	1.378 (3)
C9—N1	1.285 (3)	C18—C19	1.369 (3)
C9—O3	1.339 (3)	C18—H18	0.9300
C9—N2	1.378 (3)	C19—H19	0.9300
C10—O2	1.215 (3)	C20—O4	1.414 (3)
C10—N2	1.427 (3)	C20—H20A	0.9600
C11—N2	1.502 (3)	C20—H20B	0.9600
C11—C13	1.508 (3)	C20—H20C	0.9600
C11—C12	1.519 (3)		
			
C2—C1—C6	119.6 (2)	H12A—C12—H12C	109.5
C2—C1—C8	135.5 (2)	H12B—C12—H12C	109.5
C6—C1—C8	104.85 (19)	C11—C13—H13A	109.5
C3—C2—C1	118.2 (2)	C11—C13—H13B	109.5
C3—C2—H2	120.9	H13A—C13—H13B	109.5
C1—C2—H2	120.9	C11—C13—H13C	109.5
C2—C3—C4	120.8 (2)	H13A—C13—H13C	109.5
C2—C3—H3	119.6	H13B—C13—H13C	109.5
C4—C3—H3	119.6	C15—C14—C19	120.9 (2)
C5—C4—C3	122.1 (2)	C15—C14—O3	120.16 (19)
C5—C4—H4	119.0	C19—C14—O3	118.6 (2)
C3—C4—H4	119.0	C14—C15—C16	120.0 (2)
C4—C5—C6	116.5 (2)	C14—C15—H15	120.0
C4—C5—H5	121.7	C16—C15—H15	120.0
C6—C5—H5	121.7	C17—C16—C15	120.0 (2)
O1—C6—C5	125.7 (2)	C17—C16—H16	120.0
O1—C6—C1	111.57 (19)	C15—C16—H16	120.0
C5—C6—C1	122.7 (2)	O4—C17—C16	124.4 (2)
C8—C7—O1	112.26 (19)	O4—C17—C18	116.66 (19)
C8—C7—C10	123.7 (2)	C16—C17—C18	118.9 (2)
O1—C7—C10	123.99 (18)	C19—C18—C17	120.9 (2)
C7—C8—N1	123.5 (2)	C19—C18—H18	119.5
C7—C8—C1	106.56 (19)	C17—C18—H18	119.5
N1—C8—C1	129.93 (18)	C14—C19—C18	119.2 (2)
N1—C9—O3	121.27 (18)	C14—C19—H19	120.4
N1—C9—N2	127.03 (19)	C18—C19—H19	120.4
O3—C9—N2	111.69 (18)	O4—C20—H20A	109.5
O2—C10—C7	127.8 (2)	O4—C20—H20B	109.5
O2—C10—N2	121.9 (2)	H20A—C20—H20B	109.5
C7—C10—N2	110.30 (17)	O4—C20—H20C	109.5
N2—C11—C13	111.44 (18)	H20A—C20—H20C	109.5
N2—C11—C12	112.94 (19)	H20B—C20—H20C	109.5
C13—C11—C12	114.4 (2)	C9—N1—C8	113.92 (17)
N2—C11—H11	105.7	C9—N2—C10	121.34 (18)
C13—C11—H11	105.7	C9—N2—C11	121.94 (18)
C12—C11—H11	105.7	C10—N2—C11	116.67 (17)
C11—C12—H12A	109.5	C7—O1—C6	104.74 (16)
C11—C12—H12B	109.5	C9—O3—C14	118.74 (16)
H12A—C12—H12B	109.5	C17—O4—C20	118.16 (19)
C11—C12—H12C	109.5		
			
C6—C1—C2—C3	−1.4 (3)	C15—C14—C19—C18	0.1 (4)
C8—C1—C2—C3	178.1 (2)	O3—C14—C19—C18	173.9 (2)
C1—C2—C3—C4	0.0 (4)	C17—C18—C19—C14	0.2 (4)
C2—C3—C4—C5	1.3 (4)	O3—C9—N1—C8	−177.98 (18)
C3—C4—C5—C6	−1.1 (4)	N2—C9—N1—C8	0.8 (3)
C4—C5—C6—O1	−179.7 (2)	C7—C8—N1—C9	0.8 (3)
C4—C5—C6—C1	−0.3 (3)	C1—C8—N1—C9	179.9 (2)
C2—C1—C6—O1	−178.92 (19)	N1—C9—N2—C10	−3.9 (3)
C8—C1—C6—O1	1.4 (2)	O3—C9—N2—C10	174.94 (17)
C2—C1—C6—C5	1.6 (3)	N1—C9—N2—C11	178.7 (2)
C8—C1—C6—C5	−178.1 (2)	O3—C9—N2—C11	−2.5 (3)
O1—C7—C8—N1	179.51 (18)	O2—C10—N2—C9	−175.2 (2)
C10—C7—C8—N1	0.8 (3)	C7—C10—N2—C9	4.8 (3)
O1—C7—C8—C1	0.2 (2)	O2—C10—N2—C11	2.4 (3)
C10—C7—C8—C1	−178.48 (19)	C7—C10—N2—C11	−177.66 (17)
C2—C1—C8—C7	179.5 (2)	C13—C11—N2—C9	70.7 (3)
C6—C1—C8—C7	−1.0 (2)	C12—C11—N2—C9	−59.8 (3)
C2—C1—C8—N1	0.2 (4)	C13—C11—N2—C10	−106.9 (2)
C6—C1—C8—N1	179.8 (2)	C12—C11—N2—C10	122.7 (2)
C8—C7—C10—O2	176.5 (2)	C8—C7—O1—C6	0.7 (2)
O1—C7—C10—O2	−2.0 (4)	C10—C7—O1—C6	179.3 (2)
C8—C7—C10—N2	−3.5 (3)	C5—C6—O1—C7	178.2 (2)
O1—C7—C10—N2	177.99 (18)	C1—C6—O1—C7	−1.3 (2)
C19—C14—C15—C16	−0.4 (4)	N1—C9—O3—C14	−11.9 (3)
O3—C14—C15—C16	−174.0 (2)	N2—C9—O3—C14	169.19 (17)
C14—C15—C16—C17	0.3 (4)	C15—C14—O3—C9	−79.2 (3)
C15—C16—C17—O4	179.5 (2)	C19—C14—O3—C9	107.1 (2)
C15—C16—C17—C18	0.0 (4)	C16—C17—O4—C20	8.2 (3)
O4—C17—C18—C19	−179.8 (2)	C18—C17—O4—C20	−172.2 (2)
C16—C17—C18—C19	−0.3 (4)		

### Hydrogen-bond geometry (Å, °)

**Table:**

*D*—H···*A*	*D*—H	H···*A*	*D*···*A*	*D*—H···*A*
C4—H4···O4^i^	0.93	2.49	3.311 (3)	147
C11—H11···O2	0.98	2.25	2.761 (3)	111
C12—H12C···O3	0.96	2.32	2.845 (4)	114
C13—H13A···O3	0.96	2.41	2.957 (3)	116
C16—H16···Cg2^ii^	0.93	2.76	3.551 (2)	143
C19—H19···Cg3^iii^	0.93	2.90	3.742 (3)	152

Symmetry codes: (i) −*x*+2, −*y*+1, −*z*; (ii) *x*, −*y*−1/2, *z*−3/2; (iii) −*x*+2, *y*−1/2, −*z*+1/2.
